# Violence against women and associated factors among female construction workers in Addis Ababa, Ethiopia

**DOI:** 10.1186/s12888-023-05002-5

**Published:** 2023-07-28

**Authors:** Kidist Asratie Asegu, Anteneh Mengist Dessie, Tizita Degifie Tilahun, Gizachew Worku Dagnew, Denekew Tenaw Anley

**Affiliations:** 1 Department of Reproductive Health, Tibebe Ghion Comprehensive Specialized Hospital, Bahir Dar, Ethiopia; 2 Department of Public Health, College of Health Sciences, Debre Tabor University, Debre Tabor, Ethiopia; 3 Department of Reproductive Health and Population Studies, School of Public Health, Bahir Dar University, Bahir Dar, Ethiopia

**Keywords:** Violence, Women, Addis Ababa, Ethiopia

## Abstract

**Background:**

Violence against Women (VAW) is a global public health problem; almost one in three global women experienced one form of violence. Violence free environment is the one that everyone cherishes. However, millions of women worldwide suffer from violence. In Ethiopia, VAW is very common and considered a private matter though it has serious consequences for girls and women. Studies pointed out that it varies by workplace, and hence important to assess it among female construction workers in Addis Ababa.

**Objective:**

To assess the prevalence and factors contributing to gender-based violence on female construction workers in Addis Ababa, Ethiopia, 2021.

**Methods:**

A cross-sectional study design with a multistage cluster sampling technique was used to select 827 study participants and a face-to-face interview was held from February 24 to April 24, 2021. Data entry was done using Epi info-7 and exported to SPSS version 26 for analysis. Both bivariable and multivariable binary logistics regression analysis were employed.

**Results:**

A total of 827 female workers were interviewed in this study. The mean age of the respondents was 24.97 years with SD of ± 5.6. The magnitude of violence against female in the workplace was 70.9% (95% CI: 67.7, 73.9). This study found that females in the age group 15–19 years (AOR = 2.37, 95%CI: 1.26, 4.45), females who live in Addis Ababa for less than 3 years (AOR = 3.02, 95%CI: 1.59, 5.73) and for 3–7 years (AOR = 2.14, 95% CI: 1.14, 4.00) and females who have no formal education (AOR = 3.16, 95%CI: 1.80, 5.54) had higher odds of violence at their workplace.

**Conclusion:**

The magnitude of overall VAW among female construction workers in Addis Ababa was high compared to other workplaces. Age and the number of years lived in Addis Ababa were found to be significant factors of violence among female construction workers. Hence, emphasis shall be given for female construction workers in Addis Ababa.

## Background

The United Nations (UN) defined Gender-Based Violence (GBV) as “any act of gender-based violence that results in or is likely to result in, physical, sexual, or mental harm or suffering to women, including threats of such acts, coercion or arbitrary deprivation of liberty, whether occurring in public or private life’’ [1]. GBV occurs and is classified in numerous ways. It can be defined depending on the relationship between the perpetrator and victim (intimate partner violence (IPV) and non-IPV), or by type of the act of GBV, such as sexual, physical, or emotional violence [2].

Workplace violence is incidents where the staff is abused, threatened, or assaulted in the circumstances related to their work, including commuting to and from work, involving an explicit or implicit challenge to their safety, well-being, or health. Workplace sexual violence (WSV) is part of workplace violence that takes verbal, non-verbal, and physical forms. It can be construed as unwanted, unreciprocated, or unwelcome behavior of a sexual nature, tending to humiliate, threaten, or embarrass [3].

Violence against women (VAW) is a global public health problem [4], attributing to 197 per 1,000 deaths in 2013 alone [5], and continued being high before the COVID-19 pandemic [6]. According to a worldwide report 18 million adolescent girls aged 15–19 who had ever experienced sexual abuse, and 55 million adolescent girls in the same age group had experienced physical violence since age 15 [7].

GBV both reflects and enforces a gendered hierarchy within workplaces, and this perceived threat to the “natural” order, combined with women’s isolation, creates an atmosphere where many of these workers are subjected to abuse. Women make up only 2.6% of workers in construction and extraction occupations, and a U.S. Department of Labor study found that 88% reported experiencing sexual harassment at work [8].

Although there are some strategies and practical interventions in the global and local context, information regarding GBV against female construction workers in Ethiopia is limited [9, 10]. Some existing research was conducted focusing on violence against a female in the health sector setting and, the prevalence varies from workplace to workplace and from occupation to occupation [11]. Therefore, this study was conducted to assess the prevalence of violence and its associated factors among female construction workers.

## Methods and materials

### Study area and period

The study was conducted from February 24 –April 24, 2021, in Addis Ababa, which is the capital and largest city of Ethiopia. It is the seat of the Ethiopian federal government. As a chartered city, Addis Ababa has the status of both a city and a state. It is where the African Union is headquartered and it also hosts the headquarters of the United Nations Economic Commission for Africa (ECA), as well as various other continental and international organizations. Addis Ababa is therefore often referred to as "the political capital of Africa" for its historical, diplomatic, and political significance for the continent. The city lies a few miles west of the East African Rift valley which splits Ethiopia into two. Addis Ababa is located in the Central part of Ethiopia. The capital city, Addis Ababa is divided into ten sub-cities and 99 Kebeles. A city of more than 4 million residents, is teeming with construction activities; high-rise buildings, roads, and railways. The city saw 1,239 big constructions in the past three years, and currently, more than 5,000 big construction owners have a permit to do construction [12].

### Study design

A cross-sectional study design was applied.

### Source and study population

The source population of this study was all females employed at construction work in Addis Ababa. The study population on the other hand was female construction workers who are working in selected sub-cities.

#### Study variables

**Dependent variable**

The dependent variable was GBV (yes/no).

### Independent variables

**Socio-demographic factors** Age, educational status, marital status, income, parent’s living arrangement, parent’s educational status and parent’s occupation.

**Work-related factors** work experience, living year in Addis Ababa, working type and work time (hour) per day.

**Personal characteristics factors** Early sexual initiation, discuss reproductive health issues with friends, having social drinker friends, having Chewing chat co-workers and drinking alcohols.

#### Operational definition

**Violence against women** If women reported any of the specified acts of physical, sexual, or emotional violence committed by any individual in the workplace [13].

**Early sexual initiation** Those who have had sexual intercourse before age of 15 years [13].

**Psychological (Emotional) violence:** If women report one of the following acts: say or do something to humiliate her in front of others; threaten to hurt or harm her or someone close to her, insult her or make you feel bad about herself [13].

**Physical violence:** If women reported one of the following act: pushed her or shoved slapped her or had something thrown at you that could hurt her, beaten her, dragged her, punched her with a fist or kicked her, choked or burnt her on purpose, threaten to use or used a gun, knife or another weapon against her [13].

**Sexual violence:** If women reported one of the following act: physically force you to have sexual intercourse with him even when she did not want to; physically force her to perform any other sexual acts she did not want to; force her with threats or in any other way to perform sexual acts she did not want to [13].

### Sample size determination

The sample size was calculated by using single population proportion formula. Therefore, the sample size was determined by the formula as follows;

$$(n=\;{(Z\alpha/2)}^2\;\times p\;(1-P))/d^2$$

By taking 0.05 for margin of error (d), 50% for expected proportion (p) (for the absence of previous study done), 1.96 for Z α\2 at 95% confidence level, the calculated sample size (n) became 384. After adjusting it for the design effect, the sample size became 768. Finally, we adjusted it for 10% non-response rate and became 845.

### Sampling procedure and technique

Multistage cluster sampling techniques were employed. In the first sampling stage, three sub-cities in Addis Ababa were randomly selected using the lottery method (Lideta sub-city, Gulele sub-city, Akakikality sub-city) and four districts were selected from each selected sub-city. A total of 12 districts’ female construction workers included in the study.

### Data collection techniques

Pretested and structured questionnaire was used and the data were collected by face to face interview method. The questionnaire was first developed in English by reviewing different related works of literature and then translated to the local language (Amharic) then back translate to English to keep its consistency. Questions on socio-demographic characteristics of construction workers, magnitude and perpetrators of emotional, physical, and sexual violence, factors related to sexual, emotional, and physical violence like family characteristics, personal characteristics including construction workers' response were included in the questionnaire. A total of six (6) female data collectors with health background who work in Addis Ababa were recruited. The data collection was supervised by one senior health officer. Training was given to the field staff on the purpose of the study, principles, and ethical considerations of the data collection process. A point-by-point discussion was made on the content of the questionnaire. Questionnaires were filled by the data collectors and all data collectors were assigned to a construction site at a time to decrease information contamination. The time to complete the questionnaire was between 20 to 30 min on average and only a maximum of 10 questioners per day were filled by each data collectors. The construction workers were contacted during working hours to arrange a convenient place and time for the interview which was off-duty hours.

### Data quality control

To assure the data quality high emphasis was given to designing data collection instruments (tool). A standardized tool adapted from WHO was used. Since violence is sensitive, female data collectors were trained on the purpose, content, and ethical considerations of the study employed. Any doubts in the questionnaire were clarified by discussing each of them one by one. A pre-test was done on 42 female construction workers at the workplace before the actual data collection. Some skip patterns and questions were modified based on the result of the pre-test. During data collection, the questionnaires were reviewed for completeness, accuracy, and consistency by the supervisor every day.

### Data processing and analysis

The collected data were entered into Epi.info version 7.2 and imported to SPSS version 26 statistical software for further management and analysis. Descriptive statistics was done to describe the study population in terms of socio-demographic and other relevant variables (work-related factors, parental and personal characteristics).

To assess the association between the different predictor variables of current overall violence, first bivariate relationships between each independent variable and outcome variable were investigated using a binary logistic regression model. Those independent variables that were significant with a p-value less than 0.25 at the bivariate level were included in a multivariable analysis for the dependent variable to control for potential confounding variables. The results were presented in the form of tables and figures with their respective word description.

## Results

### Socio-demographic characteristics of participants

A total of 827 female workers were interviewed in this study with a response rate of 97.8%. About 51.9% of respondents were found to be in the age groups of 15-24 years. The mean age of the respondents was 24.97 years with SD of ± 5.6 (Table 1).

**Table 1:** Socio-demographic characteristics of female construction workers in Addis Ababa, 2021(*n* = 827)

Variables	Category	Frequency	Percentage
Age	15–19	116	14.0
	20–24	314	38.0
	25–29	247	29.9
	above 30	150	18.1
Region	SNNP	359	43.4
	Oromo	235	28.4
	Amhara	214	25.9
	Tigray	19	2.3
Religion	Orthodox	422	51.03
	protestant	302	36.51
	Muslim	63	7.62
	catholic	26	3.14
	Others	14	1.70
Marital status	Single	460	55.6
	Married	146	17.7
	Informal marriage	111	13.4
	Divorced	110	13.3
Educational status	No formal education	192	23.22
	1–8	260	31.44
	9–12	225	27.2
	Diploma and above	150	18.14
Monthly income	500–1500	86	10.4
	1501–2500	623	75.3
	2501–3500	104	12.6
	> 3500	14	1.7
Number of years lived in Addis Ababa	Below 3 years	264	31.9
	3–7 years	280	33.9
	7–10 years	100	12.0
	Above 10 years	118	14.3
	Born in Addis Ababa	65	7.9
Current living status	Alone	379	45.8
	With female friends	130	15.7
	With family	128	15.5
	With husband	124	15
	With female friends	66	8

### Personal characteristics of participants

All respondents were asked about the reasons for working as a construction worker, and more than half (58.0%) of them responded that their household low socioeconomic status is their main reason followed by the death of their parents (14.6%). Regarding their sexual experience, 488(59.8%) of the respondents had history of sexual practice (Table 2).

**Table 2:** Personal characteristics of female construction workers in Addis Ababa, 2021 (*n* = 827)

Variables	Category	Frequency	Percentage
Reasons to engage this work	Lack of income	480	58.04
	Death of family	121	14.63
	Because of profession	115	13.91
	Opposing early marriage	49	5.92
	Self-divorce	47	5.7
	Family divorce	15	1.8
History of drink alcohol	Yes	214	25.9
	No	613	74.1
History of coworker friends in the workplace chewing chat	Yes	38	4.6
	No	789	95.4
History of having sexual intercourse	Yes	488	59
	No	339	41
History of having sexual intercourse for the first time (*n* = 488)	Below 18 years	204	41.8
	Above 18 years	284	58.2
History of having coworker friend who drinks alcohol	Yes	631	76.3
	No	196	23.7
Discussion about reproductive issues with a friend (family planning, HIV/AIDS, STD)	Yes	182	22
	No	645	78

### Parental characteristics of participants

The majority of construction workers (42.9%) had both parents alive; of those 26.7% of them were living together while 16.2% of them were divorced/separated. A total of 266 (32.2%) of females lost their family by death. Regarding parental occupation, 65.7% of male parents were farmers and 41.1% of female parents were housewives followed by farmers 28.9%.

### Work-related information’s of participants

Of 827 respondents 74.6% of them were daily laborers and about 54.2% of them had 2–3 years of work experience. All of the respondents were working in the day time and only 180(21.8%) of workers had written contractual agreements.

### Magnitude of VAW

The overall magnitude of VAW was found to be 70.9% (95%CI: 67.6%, 74.1%). Emotional violence was the most prevalent (55.4%) compared with sexual and physical violence. Insulting or making feel bad about oneself accounted 296(64.2%), belittling or humiliating in front of other people accounted 262(57.8%). Regarding to perpetrators; almost all of the violence perpetrated by the employer which accounted 333(72.7%) followed by their co-workers which accounted 125(27.29%).

**Fig. 1:**
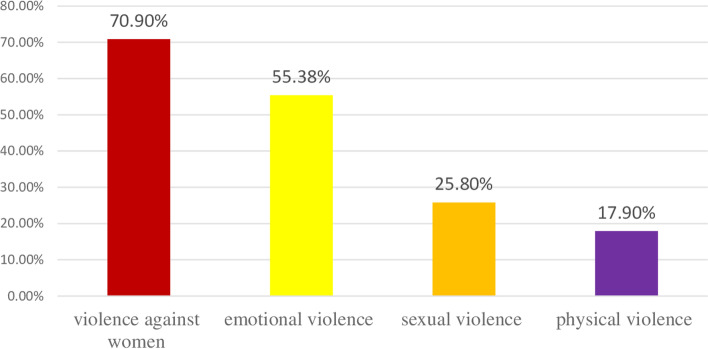
Percentage distribution of forms of violence among construction workers in Addis Ababa, Ethiopia

According to this study, the second prevalent form of VAW was sexual violence, which was reported by 211 (25.5%) study participants. Among those, 201 (95.2%) of respondents reported unwelcome sexual touch (touch on breast, genitalia, kissing…), showing porn movies, verbal jokes, or comments. A total of 34(16.1%) of construction workers reported that they were forced to have which they escaped from and 41(19.4%) faced forceful sexual intercourse. The reported perpetrators of sexual violence were co-workers 134(63.5%) followed by employers 73(34.5%) (Fig. 1).

### Why do victims not report any form of violence they faced

**Fig. 2:**
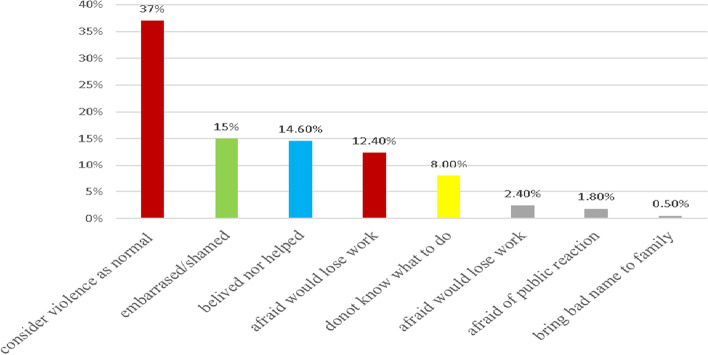
Reasons for not to report violence among female construction workers in Addis Ababa, Ethiopia

Among the victims of violence; 542(92.4%) did not report to the police, health care providers, law enforcing bodies, women’s organizations, religious leaders, family members/relatives, or peers when they encountered violence. The main reasons are illustrated by the following figure (Fig. 2).

### Factors associated with VAW

Variables like; age, number of years lived in Addis Ababa, educational status, having friends who drink alcohol, khat chewing practice of co-workers at the workplace, discussion about reproductive health issues, drinking alcohol, and working category were included in the binary logistic regression analysis, and variables with a *p*-value of < 0.25 in the binary logistic regression analysis were further considered for multivariable logistic regression. Age, educational status, and the number of years lived in Addis Ababa were found to be statically significant factors of VAW at a p-value of less than 0.05. Besides, the model fitness was assessed by Hosmer–Lemeshow test where by the *p*-value was found to be insignificant (0.072) (Table 3).

**Table 3:** Factors associated with violence against women among female construction workers in Addis Ababa, 2021

Variables	Category	VAW		COR (95% CI)	AOR (95% CI)
Age		Yes	No		
	15–19	93	23	3.092(1.71, 5.42)	2.37(1.26, 4.45)**
	20–25	241	73	2.52(1.66, 3.82)	2.24(1.41, 3.56)**
	26–30	167	80	1.59(1.05, 2.42)	2.13(1.98, 1.24)**
	Above 30	85	65	1	
History of drink alcohol	Yes	146	68	0.84(0.60, 1.18)	0.85(0.58, 1.24)
	No	440	173	1	
History of coworkers chewed chat	Yes	33	5	2.81(1.08, 7.30)	2.56(0.90, 7.32)
	No	555	236	1	
Working category	Site engineer	3	7	0.14(0.03, 0.54)	0.30(0.71, 1.32)
	Foremen	34	28	0.34(0.23, 0.67)	0.69(0.37, 1.30)
	Painter and slab worker	83	55	0.48(0.33, 0.72)	0.75(0.47, 1.19)
	Daily laborer	466	151	1	
Discuses about reproductive health issues	Yes	466	62	0.74(0.52, 1.50)	0.90(0.60, 1.34)
	No	120	179	1	
History of a friend who drinks alcohol	Yes	437	194	1.41(. 0.97, 2.03)	0.70(0.46, 1.05)
	No	149	47	1	
Educational status	No formal education	152	40	4.22(2.63, 6.78)	3.16(1.80, 5.54)**
	1–8	223	37	6.70(4.18, 10.76)	4.64(2.68, 8.02)**
	9–12	140	85	1.18(1.20, 2.78)	1.34(0.81, 2.22)**
	Diploma and above	71	79	1	
Number of years lived Addis Ababa	Below 3 years	214	50	5.31(2.98, 9.46)	3.02(1.59, 5.73)**
	3–7 years	207	73	3.52(2.01, 6.14)	2.14(1.14, 4.00)**
	7–10 years	67	33	2.52(1.32, 4.79)	1.79(0.89, 3.60)
	Above 10 years	69	49	1.74(0.94, 3.22)	1.34(0.68, 2.62)
	Born in Addis Ababa	29	36	1	

^***^=P-value < 0.001, **=*P*-value < 0.01, *=*P*-value < 0.05

## Discussion

This study was aimed to assess magnitude of violence among female construction workers and its determinant factors in Addis Ababa. The study revealed that the magnitude of violence at construction workplace was 70.9%, with emotional violence (55.38%), sexual violence (25.8%), and physical violence (17.96%). This finding was higher than studies conducted in Ethiopia; at health facilities, (29.9% and 58.2%) [14, 15], the report of the ministry of women, children and youth affairs (MOWCYA) of 2013 reported on public (56%) and private sectors (49.2%) [16], and 2016 EDHS report (23%) [13], and community-based cross-sectional studies among youths (21.5%) [17]. The possible reasons for the higher rate could be due to the difference in the study area. Relative to health care settings, the construction industry is unregulated; low average salaries, a lack of background checks prior to deployment, and physically unsound project sites and worker housing may increase the risk of violence among construction workers [8]. The national survey includes both urban and rural people, and under reporting of GBV is prominent in rural areas where women typically don't complain about men's influence. This study area difference may potentially be one of the reasons for the discrepancy seen [13, 16].

However, the prevalence of sexual violence is lower compared to a study conducted among Mekele University staff (50.2%) [15]. Construction is a high-pressure industry, thus it is likely that conflicts will arise. These conflicts can result in verbal threats, shouting, cursing, fights, raging tempers, and other violent acts. Even though there may be a large number of incidents caused by cultural obstacles and retaliation fear, sexual violence is nevertheless under reported [18]. This might be due to the construction workers' personalities and attitudes of considering violence as normal and the right of perpetrators. The other reason might be fear of openly reporting the occurrence of VAW among construction workers by using an interviewer-administered questionnaire.

The prevalence of emotional violence is in line with studies conducted in Northwest Ethiopia among housemaids (56.3%) [19]. The possible reason for this similarity may be the similarity of study participants, sample size and methods used (cluster sampling method).

It is also higher compared with studies conducted in Nigeria (52.5%) [20], and Uganda (10%) [21]. This might be due to the overall high prevalence of workplace violence in Ethiopia as reported by UNDP about gender inequality with an index value of 0.502, ranking it 121^th^ of 160 countries in 2017 [22, 23].

This study identified that females in the age groups of 15–19 are highly vulnerable than other age groups; which is in line with the national report and other studies conducted in Ethiopia (20, 43). Male dominance in the community, early adolescent physical changes, and attitudes towards violence may all play a role in the identified age group [24, 25].

The other factor of VAW was the number of years lived in Addis Ababa. Female construction workers who reside in Addis Ababa below three years were found to be 3 times more likely to experience violence when compared to other groups. Those who lived below seven years in Addis Ababa have higher risks of violence as compared to those who were born and living in Addis Ababa. This finding was consistent with a study conducted in Debre Tabor, Hadiya zone, and India. Females who were migrants from one town to another town or from rural to urban had higher odds of violence [26–28]. Internal migrants, particularly those moving from rural to urban areas, may find it difficult to find work and thus more vulnerable to violence. This is in addition to the difficulties they encounter in finding housing due to high rent rates, as well as their inability to buy food and clothing [29–32].

Educational status was significantly associated with VAW. The finding is in line with studies conducted in Mekele University female administrative staff and studies conducted in health facilities [15, 33]. In order to empower and transform women's lives, education is essential. This may be accomplished by increased social empowerment techniques like social networks, self-assurance, or the capacity to make use of the information and resources offered by society. A change in attitude and norms against violence is also encouraged by literacy [34].

Generally, this study has come up with evidences on how big VAW is in Addis Ababa construction work areas and associated factors, and pointed out that emphasis should be given from the concerned bodies. However, the study was not without limitations; there might be a social desirability bias to give accurate information since they are afraid of their employers and the cultural and normative reality of accepting violence as normal. There might also be an underestimation of physical violence because construction workers consider this violence as normal. Besides, this study was workplace-based, and there was lack of published papers on violence against women which makes difficult to compare with other studies.

## Conclusions

Despite Ethiopia is working to achieve the SDG of eliminating VAW, its magnitude among female construction workers in Addis Ababa was found to be high compared to other workplaces. Age and the number of years lived in Addis Ababa were found to be the significant factors of violence against female construction workers. Hence, Ministry of Women, Children, and Youth Affairs should give especial emphasis on violence prevention activities among female construction workers in the capital of Ethiopia.

## Data Availability

The datasets used and/or analysed during the current study available from the corresponding author on reasonable request.

## Abbreviations

CEDAW: Committee on the Elimination of Discrimination against Women
EDHS: Ethiopian Demographic and Health Survey
ETB: Ethiopian Birr
GBV: Gender-Based Violence
HIV: Human Immune Deficiency Virus
ILO: International Labor Organization
IPV: Intimate Partner Violence
MOWCYA: Ministry of Women, Children and Youth Affairs
SDG: Sustainable Development Goals
SPSS: Statistical package for social sciences
STD: Sexual Transmitted Disease
UDHR: Universal Declaration of Human Right
UN: United Nation
UNICEF: United Nation Children’s Emergency Fund
VAW: Violence against Women
WHO: World Health Organization
